# Global Transcriptomic Responses of *Roseithermus sacchariphilus* Strain RA in Media Supplemented with Beechwood Xylan

**DOI:** 10.3390/microorganisms8070976

**Published:** 2020-06-29

**Authors:** Kok Jun Liew, Neil C. Bruce, Rajesh Kumar Sani, Chun Shiong Chong, Amira Suriaty Yaakop, Mohd Shahir Shamsir, Kian Mau Goh

**Affiliations:** 1 Faculty of Science, Universiti Teknologi Malaysia, Johor 81310, Malaysia; kokjunliew@gmail.com (K.J.L.); cschong@utm.my (C.S.C.); shahir@utm.my (M.S.S.); 2 Centre for Novel Agricultural Products, Department of Biology, University of York, Wentworth Way, York YO10 5DD, UK; neil.bruce@york.ac.uk; 3 Department of Chemical and Biological Engineering, South Dakota School of Mines and Technology, Rapid City, SD 57701, USA; rajesh.sani@sdsmt.edu; 4 School of Biological Sciences, Universiti Sains Malaysia, Pulau Pinang 11800, Malaysia; amirasuriaty@usm.my; 5 Faculty of Applied Sciences and Technology, Universiti Tun Hussein Onn Malaysia, Pagoh Higher Education Hub, Johor 84600, Malaysia

**Keywords:** *Rhodothermaceae*, *Roseithermus*, RNA-Seq, lignocellulolytic, CAZymes, xylanase

## Abstract

The majority of the members in order *Rhodothermales* are underexplored prokaryotic extremophiles. *Roseithermus*, a new genus within *Rhodothermales*, was first described in 2019. *Roseithermus sacchariphilus* is the only species in this genus. The current report aims to evaluate the transcriptomic responses of *R. sacchariphilus* strain RA when cultivated on beechwood xylan. Strain RA doubled its growth in Marine Broth (MB) containing xylan compared to Marine Broth (MB) alone. Strain RA harbors 54 potential glycosyl hydrolases (GHs) that are affiliated with 30 families, including cellulases (families GH 3, 5, 9, and 44) and hemicellulases (GH 2, 10, 16, 29, 31,43, 51, 53, 67, 78, 92, 106, 113, 130, and 154). The majority of these GHs were upregulated when the cells were grown in MB containing xylan medium and enzymatic activities for xylanase, endoglucanase, β-xylosidase, and β-glucosidase were elevated. Interestingly, with the introduction of xylan, five out of six cellulolytic genes were upregulated. Furthermore, approximately 1122 genes equivalent to one-third of the total genes for strain RA were upregulated. These upregulated genes were mostly involved in transportation, chemotaxis, and membrane components synthesis.

## 1. Introduction

Lignocellulosic biomass consists of lignin, cellulose, and hemicellulose. In nature, microbial consortia rather than a single taxon biodegrade plant biomass [1]. Individual members in the consortia often have specific roles and preferences toward the lignocellulolytic carbohydrates such as cellulose and xylan [2]. *Asticcaculis*, *Cellulomonas*, and *Chryseobacterium* are good cellulose degraders; however, they are ineffective at degrading hemicellulose [2]. *Caulobacter*, *Sphingobacterium*, and *Sphingobium* hydrolyze hemicellulose but are inefficient at hydrolyzing cellulose [2]. Not many bacteria produce ligninolytic enzymes; examples of bacteria with that ability are *Pseudomonas* and *Sinorhizobium* [3,4]. Lignocellulolytic microorganisms have been isolated from a diverse range of isolated environments, including soils, compost, aquatic habitats, digestive tracts of herbivores, termite guts, landfill, and waste digesters [1]. Among the many, *Caldicellulosiruptor*, *Rhodothermus*, and *Pseudoxanthomonas* are effective degraders of cellulose and hemicellulose [5,6,7].

Glycoside Hydrolases (GHs) constitute a large group of hydrolase enzymes. The Carbohydrates-Active Enzyme (CAZy) classifies GH families based on protein sequence similarities. Currently, 167 GH families are documented in the CAZy database [8]. Cellulases are a group of proteins comprising endoglucanases, exoglucanases, and β-glucosidases [9]. These enzymes act synergistically to achieve the complete degradation of cellulose. Endoglucanases, or also known as endo-1,4-β-d-glucanases (EC 3.2.1.4), are classified in GH 5, 6, 7, 8, 9, 10, 12, 26, 44, 45, 48, 51, 74, and 124 [8]. Endoglucanases initiate the cellulose hydrolysis process by acting on an amorphous region of the cellulose and yield cellodextrins with different lengths [10]. β-glucosidases (EC 3.2.1.21) are involved in the final step of cellulose saccharification by hydrolyzing β-1,4-glycosidic linkages of the cellobiose and produce glucose. Due to the high complexity of the hemicellulose structure, several types of enzymes are required to completely hydrolyze this polymer. For instance, xylanase, β-xylosidase, α-l-arabinofuranosidase, acetyl xylan esterase, ferulic/coumaric acid esterase, and α-glucuronidase are major enzymes required for hemicellulose degradation [11]. Xylanases, also known as endo-1,4-β-xylanase (EC 3.2.1.8), are responsible for the cleavage of β-1,4-xylosidic linkages in hemicellulose backbone. β-xylosidases, also known as xylobiases (EC 3.2.1.37), break the β-1,4-xylosidic linkages in xylobioses or other xylooligosaccharides and produces xylose as the end products.

*Rhodothermales* have been organized into four families: *Rhodothermaceae*, *Salinibacteraceae*, *Salisaetaceae*, and *Rubricoccaceae*, which are represented by a total of 10 genera [12,13]. Family *Rhodothermaceae* consists of genera *Rhodothermus* and *Roseithermus* [7,13]; *Salinibacteraceae*–*Salinibacter* and *Salinivenus* [14,15]; *Rubricoccaceae*–*Rubricoccus* and *Rubrivirga* [16,17]; *Salisaetaceae*–*Salisaeta*, *Longimonas*, *Longibacter*, and *Natronotalea* [18,19,20,21]. Most of them are underexplored polyextremophiles [12,13,22]. Table 1 provides some information on these *Rhodothermales* members. All of them are halophiles, as they were isolated from salty environments such as marine and saltern lakes. Members of *Rhodothermaceae* are halothermophile. *Rhodothermus* is well understood from the aspects of phenotypic, chemotypic, genomic and extrachromosomal elements, pathway and gene manipulations, environmental adaptation, as well as the discovery of industrial enzymes [23,24,25,26,27]. Bacteria strains such as *Rhodothermus marinus* (DSM 4252) and *R. marinus* SG0.5JP17-172 are excellent plant biomass degraders [28,29]. In contrast, other genera in the *Rhodothermales* order have not been examined for lignocellulosic degradation.

*Roseithermus sacchariphilus* strain MEBiC09517^T^, the only type strain of the genus *Roseithermus* is a thermophile that grows optimally at 55 °C and pH 7 [13]. This bacterium was isolated from a seawater sample obtained at a seaport near a wood processing factory located in South Korea [13]. The understanding of strain MEBiC09517^T^ is limited to phenotypic and chemotypic data. The bacterium can utilize different substrates such as carboxymethyl cellulose, cellobiose, pectin, maltose, and mannose; however, the ability to use starch, xylan, arabinan, xylose, arabinose, etc. was not reported [13]. Besides, lignocellulosic degradation and carbon utilization capabilities of the *R. sacchariphilus* strain MEBiC09517^T^ are yet to be further studied and reported.

*Roseithermus sacchariphilus* strain RA studied in this work was isolated in 2014 from the Ayer Hangat hot spring located in Langkawi Island, Malaysia [30,37]. The complete genome of strain RA was sequenced earlier [30,38]. When this bacterium was initially isolated, it was not assigned to any genera due to low 16S rRNA sequence similarity (<90%). The closest taxa were those from the *Rhodothermaceae* family. Hence, the bacterium was tentatively designated as *Rhodothermaceae* bacterium RA. Now, the bacterium has been renamed as *R. sacchariphilus* strain RA due to its high 16S rRNA and genome-to-genome similarity to a newly proposed type strain *R. sacchariphilus* strain MEBiC09517^T^ in 2019 [13] (Table 1). Our team has examined strain RA from multiple aspects to understand this underexplored genus [22]. Based on the genome annotation, strain RA harbors two non-homologous xylanases, XynRA1 and XynRA2. Both proteins exhibit slightly different in vitro biochemical characteristics [31,32]. Additionally, strain RA expresses up to four non-homologous cellulases, two β-glucosidases, and four α-l-rhamnosidases [38]. The biological functions of these non-homologous glycosyl hydrolases are divergent. We anticipated that the cultivation conditions would influence the expression levels of these proteins. This is the first report describing the global cell responses and expression of glycosyl hydrolase genes when the bacterium *R. sacchariphilus* strain RA is cultivated in a medium supplemented with xylan.

## 2. Materials and Methods

### 2.1. Bacterial Strain

*R. sacchariphilus* strain RA was deposited in the Korean Collection of Type Cultures with the assigned number KCTC 62031. The bacterium was grown on Marine Agar (MA) or in Marine Broth (MB, Laboratories CONDA, Madrid, Spain) at pH 7.5 and temperature 50 °C.

### 2.2. Growth Profile

Strain RA grown in MB was treated as the control experiment. Commercially available MB powder consists of yeast extract and peptone that contain traces of organic carbon or carbohydrates [39]. The bacterium was separately cultured in MB enriched with 0.1% (*w*/*v*) beechwood xylan (Megazyme, Wicklow, Ireland). The optical density (OD_600nm_) of each culture was determined using a 7300 visible spectrophotometer (Jenway, Stone, UK), and CFU/mL was determined for six days (144 h). CFU was calculated by cell counting after spreading the cultures on Marine Agar and incubated for 48 h at pH 7.5 and 50 °C. The growth profile analysis was conducted in triplicate.

### 2.3. Enzyme Activities

Cultures were harvested from each flask and centrifuged at 8000× *g* for 5 min. Unless specified, centrifugation was done at 4 °C using a tabletop Eppendorf 5427R refrigerated centrifuge (Eppendorf, Hamburg, Germany). A cell-free supernatant of each culture was assayed for xylanase, endoglucanase, β-xylosidase, and β-glucosidase. The modified dinitrosalicyclic acid (DNS) assay with 1% (*w*/*v*) beechwood xylan or CMC was used to determine the xylanase and endoglucanase activity, respectively. The protocol for the DNS assay was conducted according to Kahar et al. [40]. In brief, the reaction mixture consisted of 500 μL of substrates and 50 μL of enzyme samples and was incubated at 50 °C, pH 8 for 15 min. One unit (U) of xylanase or endoglucanase activity is defined as the amount of enzyme that catalyzed the formation of 1 μmol reducing sugar (xylose or glucose) per min per mL under the assay conditions. The activity of β-xylosidase and β-glucosidase was determined using 5 mM of synthetic substrates *p*-nitrophenyl-β-d-xylopyranoside (*p*NPX) and *p*-nitrophenyl-β-d-glucopyranoside (*p*NPG) (Merck Millipore, Burlington, NJ, USA), respectively [41,42]. One unit (U) of β-xylosidase or β-glucosidase activity is defined as the amount of enzyme that catalyzed the formation of 1 μmol of *p*-nitrophenol (*p*NP) per minute per mL under the assay conditions (50 °C, pH 8). All the enzyme assays were performed in triplicate.

### 2.4. RNA Extraction

Total RNA extraction was performed after cultivating *R. sacchariphilus* strain RA on MB and MB+xylan, respectively. Cell pellets were collected by centrifugation (4 °C, 5000× *g*, 10 min). Unless stated, all the following steps were conducted on ice and under RNase-free conditions. The harvested cells were resuspended in 1× DNA/RNA shield solution, and the mixtures were transferred to ZR BashingBead^TM^ Lysis Tubes consisted of a combination of 0.1 mm and 0.5 mm beads (Zymo Research, Irvine, CA, USA). The cells were lysed by the bead beating method in a TissueLyser II (Qiagen, Hilden, Germany) at 2 × 20 hertz for 3 min. Then, the cell debris was discarded by centrifugation at 10,000× *g* for 5 min. The supernatant liquid was subjected to purification by using a Quick-RNA Miniprep Plus Kit (Zymo Research, Irvine, USA). A DNase-I treatment was also included during the purification to remove traces of DNA. The extracted RNAs were quantified by a NanoDrop^TM^ 1000, Qubit 3.0 Fluorometer coupled with RNA HS Assay Kit (Thermo Fisher Scientific, Waltham, USA) and on a 1% (*w*/*v*) agarose gel. Bioanalyzer 2100 accompanied with an RNA 6000 Nano Assay kit and chips (Agilent Technologies, Santa Clara, CA, USA) were used to determine the RNA integrity number (RIN) of the extracted RNA. The experiments involved at least three biological replicates.

### 2.5. Library Preparation and RNA Sequencing

RNA library preparation and sequencing were performed by Novogene Co., Ltd. (Beijing, China). Triplicate samples were subjected to rRNA depletion using the protocols of Ribo-Zero rRNA Removal Kit (Illumina, San Diego, CA, USA). Then, the purified rRNA-depleted samples were fragmentized and utilized as the template for cDNA synthesis via reverse transcription. The cDNA was subjected to library preparation and multiplexing using a TruSeq stranded mRNA Library Prep Kit and TruSeq RNA CD Index Plate (Illumina). The quality of the prepared library was verified using a Bioanalyzer 2100. RNA sequencing was carried out using setting PE 150 (paired-end read 150 bp) in HiSeq4000 (Illumina). A minimum of 20 million reads was reserved for every sample.

### 2.6. Data Processing and Differentially Expressed Genes (DEGs) Analysis

The resulting raw sequence reads generated from the sequencer were subjected to data quality control. Trimmomatic v0.36 with a default parameter was used for error rate assessment and poor reads filtering [43]. Mapping of the clean reads to the complete genome of *R. sacchariphilus* strain RA (Accession number: CP020382) was performed using Bowtie2 software [44]. Expression levels of individual genes were determined by counting the reads that were mapped to the complete genome. The FPKM approach (Fragment Per Kilobase of transcript sequence per Millions base-pair sequenced) was used to facilitate differential expression analysis (DEGs) between the control and beechwood xylan setups. Fold change (FC) is calculated by dividing the normalized read counts of a gene at one condition (also known as the case) to the read counts of the same gene at another condition (also known as the control or reference). The ‘case’ in this article refers to MB+xylan, while the ‘reference’ refers to MB alone. Besides setting an FC cut-off point, a statistical parameter—the false discovery rate (FDR)—is used to identify the false-positive result. FDR uses padj (adjusted p-value) as an indicator [45]. The cut-off value for padj set in this study is 0.05. A similar threshold was adapted in other articles [46,47]. If the padj is > 0.05, the FC is considered as statistically insignificant, or it is also known as a false-positive DEG. In this study, a true DEG is acknowledged with a fold change of 1.5 and shall be statistically significant (padj is < 0.05).

HTSeq was used to analyze the gene expression levels using union mode [48], DESeq v1.10.1 was used for DEGs analyses [45], GOSeq was used for Gene Ontology (GO) Enrichment analysis [49], and KOBAS v3.0 was used for Kyoto Encyclopedia of Genes and Genomes (KEGG) analysis [50]. All the raw sequence data from the RNA-seq have been deposited in The National Center for Biotechnology Information (NCBI) Sequence Read Archive (SRA) database with the SRA accession numbers SRX6798561–SRX6798566.

## 3. Results and Discussion

### 3.1. Growth Profiles

**Figure 1:**
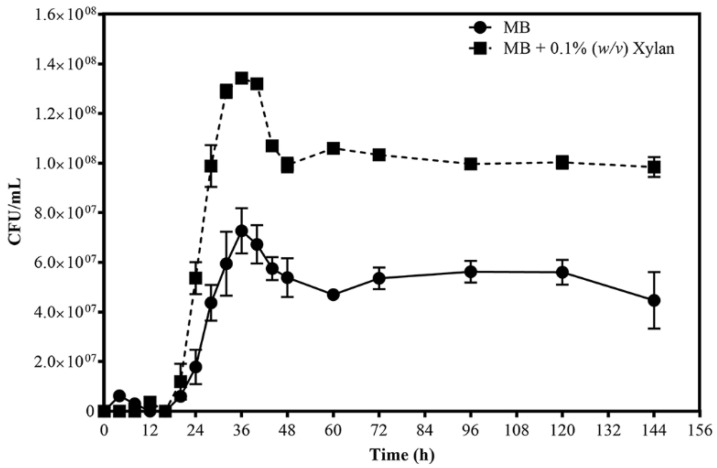
Growth profile of *R. sacchariphilus* strain RA grown on Marine Broth (MB) and MB supplemented with beechwood xylan (0.1% *w*/*v*).

*R. sacchariphilus* strain RA was cultured for six days, in either MB or MB+xylan. Figure 1 shows the growth profiles. The patterns for growth on each medium are quite similar, with a lag phase of approximately 24 h, followed by an exponential phase (24–36 h), and eventually CFU decline after that (36–48 h) before plateauing. When cultivating in MB and supplemented MB media, strain RA achieved maximum growth after 36 h, and the cell concentration was almost double in MB+xylan (approximately 1.34 × 10^8^ CFU/mL) compared to growth in MB (approximately 7.3 × 10^7^ CFU/mL). At the exponential phase, the growth rate and doubling time (T_d_) cultivated on MB+xylan were 0.28 h^−1^ and 106 min, respectively, whereas they were 0.25 h^−1^ (growth rate) and 122 min (T_d_) for the bacteria grown on MB. The enzymatic hydrolysis of xylan yielded xylose and xylooligosaccharides; these additional carbon sources affected the growth of strain RA. Collectively, the data showed that strain RA could grow better when xylan was available.

### 3.2. Enzyme Activities

**Figure 2:**
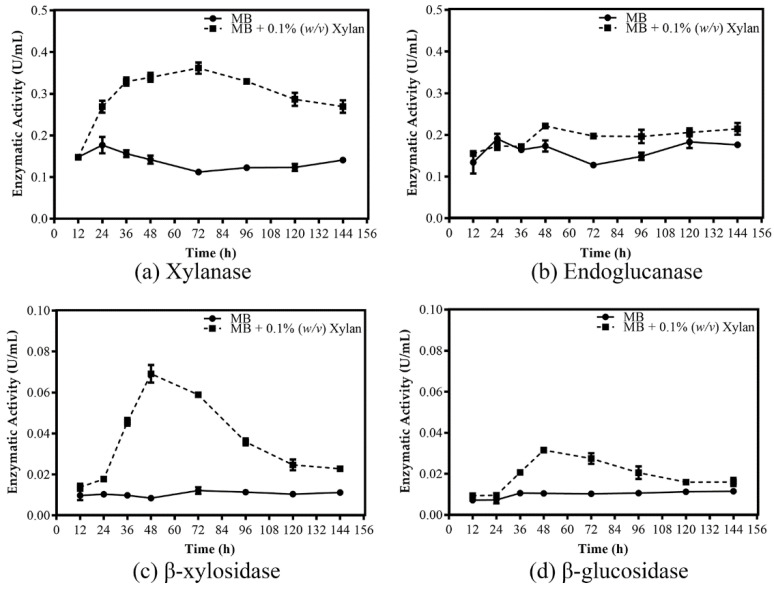
Enzyme activity of *R. sacchariphilus* strain RA crude lysate throughout its growth profile. (**a**) Xylanase; (**b**) endoglucanase; (**c**) β-xylosidase; (**d**) β-glucosidase activities.

Enzyme activities (xylanase, endoglucanase, β-xylosidase, and β-glucosidase) of strain RA growing in MB and MB+xylan were determined (Figure 2). Endoglucanase and xylanase are primary enzymes for hydrolyzing cellulose and xylan in hemicellulose, respectively. β-xylosidase and β-glucosidase are pivotal enzymes in the saccharification process in forming monomeric sugars. Detected enzyme activities were low throughout the six days (144 h) if cells were cultivated in MB alone. Xylanase is induced to a maximum of 3-fold at 72 h but then declines to a small extent thereafter (Figure 2a). β-xylosidase and β-glucosidase are induced 8-fold and 3-fold, respectively, at 48 h, but both then decrease significantly after that (Figure 2c,d). In contrast, endoglucanase activity is induced to a much less significant extent and shows no corresponding decline to that recorded for the other three enzymes (Figure 2b).

### 3.3. Technical Overview of RNA-Seq Data

**Figure 3:**
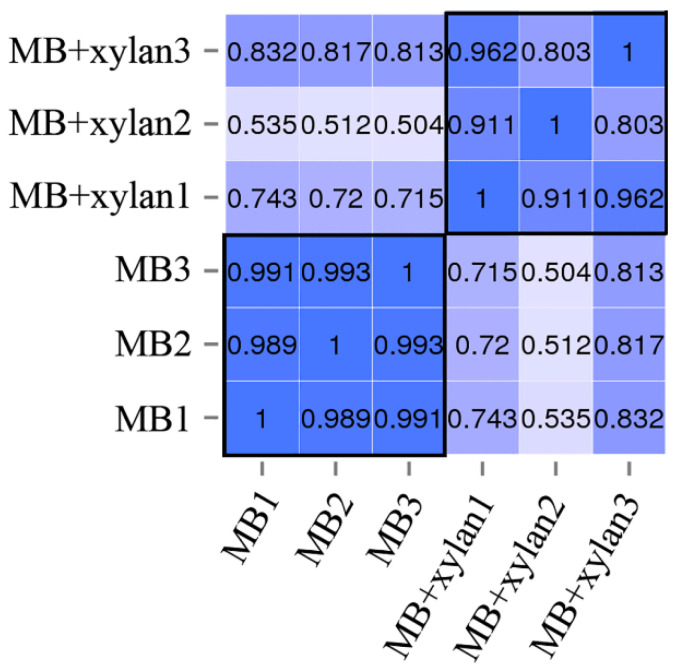
The correlation (R^2^ value) between the samples. 1, 2, and 3 represent the three biological replicates of each experiment. MB: *R. sacchariphilus* strain RA culturing on MB medium. MB+xylan: Bacteria growth on MB supplemented with beechwood xylan (0.1% *w*/*v*).

The total RNA-extraction harvesting time was 36 h when *R. sacchariphilus* strain RA had achieved maximum CFU in MB and MB+xylan (Figure 1). Based on the enzyme activity time plots (Figure 2), the marker enzymes reached maximum activities at 48 h. Therefore, harvesting cells after 36 h is reasonable, as most of the RNA transcripts for (hemi-)cellulolytic genes would still be intact. Several attempts were made to harvest the total RNA in earlier cultures (< 36 h); however, the concentration of extracted RNA was too low. The RNA integrity number (RIN) for all the samples ranged from 6.3 to 8.0. The samples also passed other quality-control (QC) requirements for RNA sequencing. Table 2 summarizes the QC statistics information for each replicate after sequencing. Around 21–25 million raw reads were generated for each sample. The raw reads were filtered using Q_Phred_20 (Q20). After filtering low-quality reads, each sample had 20–24 million good reads. Approximately 99.51% (for MB samples) and 98.76% (MB+xylan) of the total clean reads could be aligned with the genome of *R. sacchariphilus* strain RA. Multiple mapped reads accounting for <1.5% only. Collectively, the quality of sequencing and mapping of the reads is reliable. Figure 3 shows the squared value of the Pearson correlation coefficient (R^2^) between the samples. This value is a critical evaluating indicator to test the reliability and reproducibility of the experiment [51]. The tolerable range of R^2^ between the bioreplicates should be greater than 0.8 [52]. In this study, the R^2^ values for the three replicates of all settings exceeded 0.8. This means that the data across replicates are reliable and reproducible.

A fold change (FC) of 2.0 is a commonly used threshold for differential expression analysis (DEG). In this study, a gene is acknowledged as true DEG if the FC is 1.5 and statistically significant with a padj lower than 0.05. As stated earlier, xylanase activity was significantly enhanced in MB+xylan compared to that of MB alone. Many essential glycosyl hydrolase genes—for instance, the xylanase genes—had an FC less than 2. Furthermore, genes related to transcriptional machinery, chemotaxis, cell motility, and carbon metabolism generally have FC in the range of 1.5 to 2.0. However, if the cut-off value was set as two folds in this study, the threshold is too high and unreliable to manifest the nature of the current experimental data.

### 3.4. Overview of Differential Expression (DEGs) Analysis

**Figure 4:**
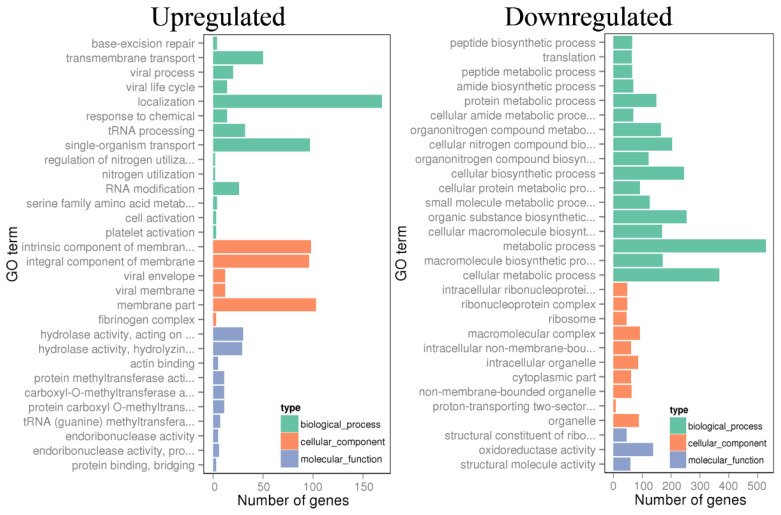
GO Enrichment analysis of the up- and downregulated gene pools of strain RA cultivated on beechwood xylan.

DEGs analysis interprets the changes in transcript abundance [53]. Figure 4 shows the overall gene expression profile of strain RA grown in MB+xylan. The total number of upregulated genes was 1122 out of 3700 protein-encoding genes in the genome. Based on the GO-Enrichment analysis, the majority of the upregulated genes were involved in transportation, membrane components synthesis, and hydrolase enzymes production (Figure 4). The total number of downregulated genes were 1039, and these were mainly engaged in metabolic processes and various biosynthesis pathways.

### 3.5. Transcription Factors

The regulation of transcription involves transcriptional machinery (RNA polymerase subunits, sigma factors (σ), and other regulatory factors) in addition to transcriptional factors (TFs). TFs are DNA-binding proteins that affect the action of RNA polymerase [54]. *R. sacchariphilus* strain RA harbors several AraC family TFs (AFTR), and these proteins are encoded by gene ID AWN76_004845, 005395, 009110, 012545, and 014955. In general, AFTR is well studied using *E. coli* models and is involved in regulating genes for carbon catabolism pathways, stress responses, or virulence [55]. Members of AFTR are known to be very diverse and primarily function as transcriptional activators [56]. Most of the AFTR in strain RA were not responsive or downregulated, except for AFTR AWN76_005395 (Table 3). This upregulated protein has a transcription regulator helix-turn-helix (HTH) domain at the N-terminal region. It is understood from earlier work that AFTR proteins use the HTH domain to sense and interact with effector molecules, such as l-arabinose or other similar sugars [57]. Other TF families such as DeoR, LacI, and GntR are also known to regulate carbon metabolism and sugar transportation [54], and these TFs are usually repressors [58,59,60]. The TFs in these families were identified in strain RA transcriptome datasets (Table 3). Among these up- and downregulated TFs, the LacI TFs (AWN76_006400, 004120, 013990) and GntR TF AWN76_008220 were located adjacent to some clusters of (hemi-)cellulolytic GHs. Thus, these four TFs are most probably crucial in regulating the expression of the GHs and affecting the hydrolysis of the beechwood xylan in this study. The upregulation of activators LacI AWN76_006400, 004120, and 013990 led to the upregulation of the nearby GHs genes. In contrast, GntR TF AWN76_008220 is probably a repressor, as its downregulation led to the upregulation of the neighboring GHs.

### 3.6. Chemotaxis and Cell Motility Response

**Figure 5:**
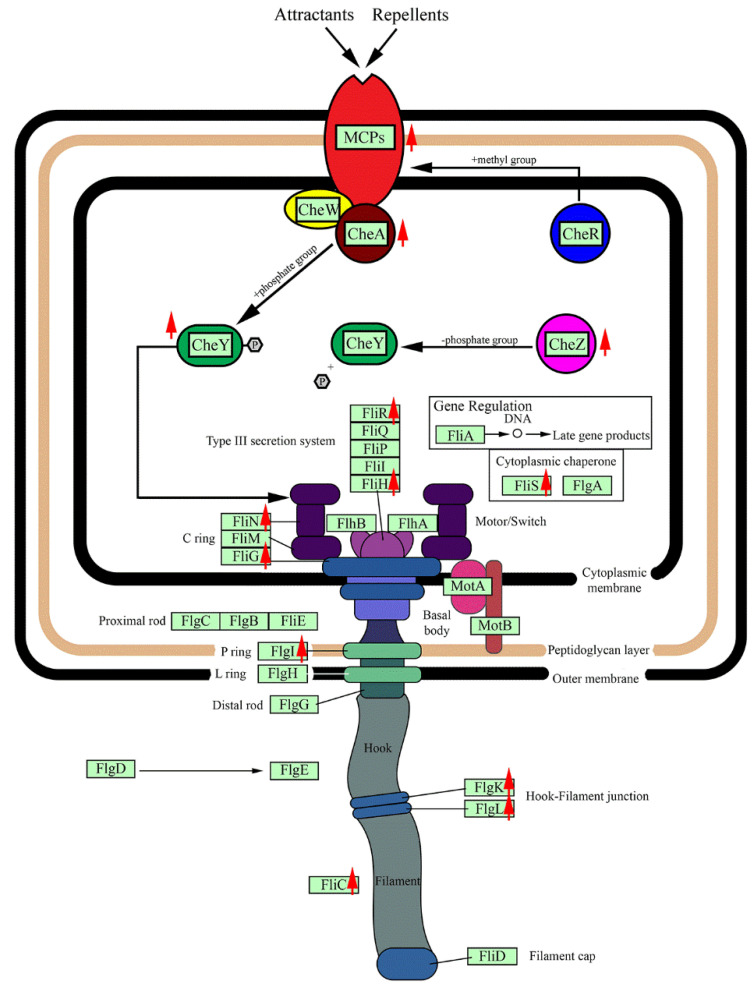
Schematic diagram for chemotaxis pathway and flagella assembly in strain RA. The red arrows indicate the upregulated genes when strain RA grows on MB supplemented with beechwood xylan.

Chemotaxis is the directed motion of a microorganism that involves signaling and movement by flagella. The chemotaxis pathway involves multiple genes; these genes in the *R. sacchariphilus* strain RA are MCP, CheA, CheR, CheW, CheY, and CheZ (Table 4, Figure 5). Methyl-accepting chemotaxis protein (MCP) is a transmembrane sensor protein that receives signals from the environment [61]. In the genome of *R. sacchariphilus* strain RA, five different MCP-encoding genes were identified (AWN76_009955, 011645, 016375, 017480, and 017530). Among these, three out of five were upregulated when strain RA was supplemented with xylan (Table 4). Xylose or other sugars from the degraded xylan are possibly the signal molecules sensed by the upregulated MCPs.

CheA is a signal transduction histidine kinase that mediates chemotaxis responses by phosphorylating CheY [62]. In *R. sacchariphilus* strain RA, CheA (AWN76_017495) was upregulated (Table 4). These data indicate that the addition of xylan may increase the chemotaxis response of strain RA to a greater extent to that of MB alone. CheY is also a chemotaxis response regulator [63]. Two CheY genes in strain RA—AWN76_008370 and 016800—were highly upregulated (Table 4). In many prokaryotes, phosphorylated-CheY binds to the flagellar motor switch protein and induces cell movement by the changes of flagellar rotation [62]. The flagellar motor switch proteins of strain RA are comprised of FliM, FliG, and FliN, and many of the genes related to these proteins were upregulated. Other genes related to flagellar function were also upregulated in the experiment (Table 4, Figure 5). A portion of the genes related to chemotaxis and flagella assembly were downregulated or less responsive to the additional of xylan.

### 3.7. CAZymes in R. sacchariphilus Strain RA

**Figure 6:**
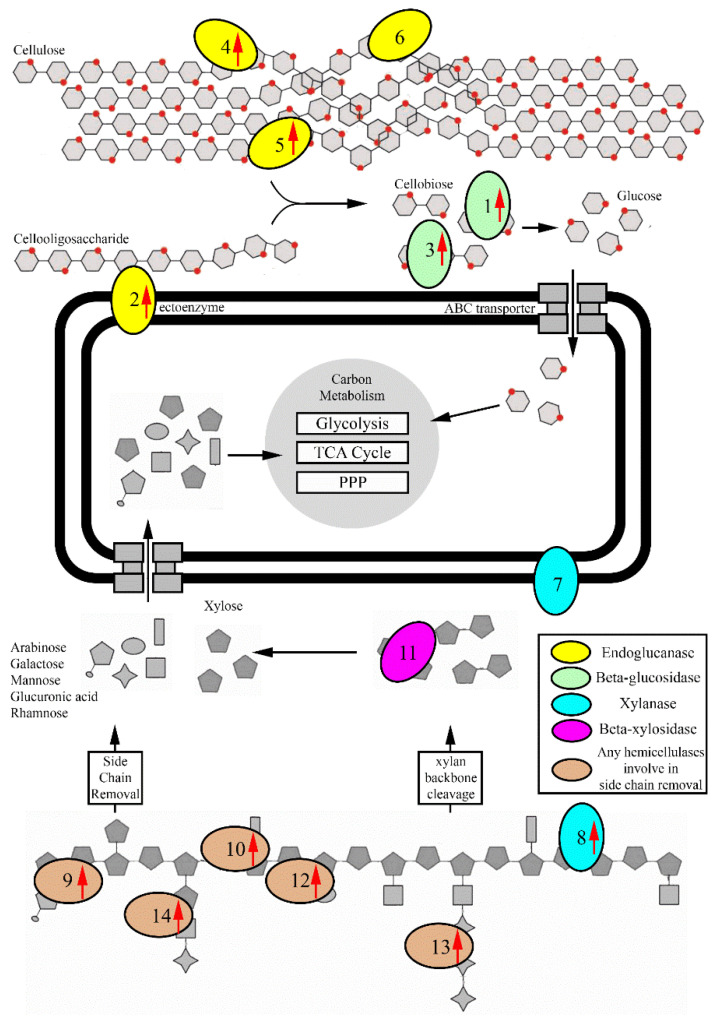
Illustration of (hemi-)cellulolytic hydrolysis of strain RA. 1–14: Selected enzymes listed in Table 5. Please refer to Table 5 for the complete protein names, family, and fold change of each enzyme. The red arrows indicate the upregulated genes when strain RA grows on MB supplemented with beechwood xylan.

CAZymes is a generic name referring to carbohydrate-acting enzymes listed in the CAZy database, which includes the glycoside hydrolase family of proteins (GHs) and auxiliary-activities enzymes (AAs). To identify how the *R. sacchariphilus* strain RA degrades lignocellulosic biomass, mining of the genes that encode for GHs and AAs was conducted in silico using dbCAN HMMs version 5.0 [64]. A total of 54 GHs that affiliated with 30 families were identified in the strain RA’s genome. These families include GH2, 3, 5, 9, 10, 13, 15, 16, 18, 20, 23, 24, 29, 31, 33, 43, 44, 51, 53, 67, 77, 78, 88, 92, 106, 113, 130, 140, 144, and 154. Six genes associated with GH3, 5, 9, and 44 were related to cellulose degradation, and they shared low amino acid sequence identities to other counterpart sequences available in the NCBI database (57%–73% identity). Strain RA has 20 genes related to hemicellulose degradation (Table 5, Figure 6). These hemicellulose-acting enzymes belong to GH 2, 10, 16, 29, 31, 43, 51, 53, 67, 78, 92, 106, 113, 130, and 154. These enzymes also shared low similarity with the existing sequences deposited in databases (57%–72% identity). For example, endo-1,4-β-xylanase (encoded by AWN76_003690) and β-xylosidase (AWN76_008215) are 72% in similarity with the counterpart enzymes harbored in the members in *Rhodothermales* order– *Rhodothermus marinus* and *Rubrivirga marina*, respectively. The α-mannosidase (AWN76_009395) shares a low similarity of 57% to that of enzyme produced by *Pedobacter ginsengisoli*.

The induction of hemicellulolytic genes was observed when beechwood xylan was added to the culture medium. Out of the 20 hemicellulolytic genes present in the genome, 11 were upregulated. For example, the endo-1,4-β-xylanase (AWN76_008205), a primary hemicellulose backbone cutter in strain RA was upregulated (Table 5, Figure 6). Moreover, the response level was higher for other hemicellulolytic enzymes acting on the hemicellulose side-chain cleavage. For instance, the β-mannase (AWN76_013895) and two glycosidases (AWN76_014035 and 014055) are putatively involved in mannan degradation and mannose side-chain hydrolysis. Other CAZymes—for example, α-l-rhamnosidase, α-glucuronidase, α-n-arabinofuranosidase, and endo-1,4-β-galactanase were also upregulated. These enzymes can putatively degrade polymers or side chains made up of arabinose, galactose, and rhamnose sugar monomers [6,65,66].

The initial design of the experiment has one potentially significant limitation as we hypothesized that the addition of xylan (representing hemicellulose) in the medium would enhance only hemicellulases and not cellulases. However, the data clearly shows that strain RA’s cellulolytic enzymes are readily induced with the introduction of xylan. Five out of six cellulolytic genes were upregulated, with the FC range of 1.45–5.30 (Table 5, Figure 6). In the natural environment, pure cellulosic or hemicellulosic polymers rarely exist individually. Instead, the two polymers co-exist as part of the whole plant biomass or fragments of particulate carbon sources. It is relatively hard to cleave cellulose to form hydrolysate cellodextrins or glucose, as the polymer is recalcitrant to degradation, and its solubility is relatively low [51]. It is energy-expensive for a microorganism to trigger cellulase expression by a single route using hydrolysates generated from cellulose–polymer breakdown [67,68]. To overcome the limitation stated above, certain bacteria can use hemicellulose or other monomeric sugars (xylose, galactose, arabinose, etc.) to induce the formation of both cellulases and hemicellulases [69,70,71]. In this study, strain RA might exhibit the same characteristics as other bacteria, where cellulolytic enzymes of strain RA are more readily induced by hemicellulose, xylan, monomer sugars (i.e., xylose), or xylan hydrolysates. In strain RA, the cellulolytic genes (i.e., AWN76_008195, 008215, and 008290) are located close to other hemicellulolytic genes (i.e., AWN76_008205, 008230, and 008320). The same promoter and GntR transcription factor (AWN76_008220) probably control the expression of these neighboring genes.

Based on the genome annotation, strain RA can express up to four non-homologous cellulases (FC 1.5–2.0), two β-glucosidases (FC 3.4 and 5.30), four α-l-rhamnosidases (FC 1.6–3.3), two xylanases, and other multiple non-homologous proteins. Strain RA is a halo-thermophile. The majority of strain RA’s proteins are likely to be thermostable and salt-tolerant to ensure all biological pathways are functional in high salt. So far, only two xylanases from this bacterium have been characterized [31,32]. Interestingly, xylanase XynRA1 (AWN76_008205, upregulated in MB+xylan) functions better in low salinity, while XynRA2 (AWN76_003690, non-DEG xylanase) exhibits a higher salt tolerance up to 5.0 M NaCl. Both enzymes displayed different characteristics, including product specificity, kinetic performance, and responded differently to temperature, pH, substrate, and metal ions [31,32].

In general, the AAs are redox enzymes that can assist and work simultaneously with other CAZymes to saccharify plant biomass [8]. Examples of AAs include ligninolytic enzymes and lytic polysaccharide mono-oxygenases (LPMOs) [8,72]. The genome of *R. sacchariphilus* strain RA may harbor 8 putative AAs based on the prediction by dbCAN HMMs 5.0 (Table 6) [64]. Out of these 8 AAs, only two were upregulated, other AAs were either downregulated or non-responsive. These upregulated genes belong to the AA3 family. The AA3 family is involved in the oxidation of carbohydrates, alcohols, unsaturated alcohols, and branched-chain or secondary alcohols [73]. In this experiment, these two AAs may probably assist other GHs of the bacterium to degrade the beechwood xylan. However, the biochemical functions of these putative AAs proteins are yet to be determined.

### 3.8. Sugar Transportation and Carbon Metabolism in R. sacchariphilus Strain RA

When required or sensed by the bacterium, strain RA will import sugars or simple saccharides via various transporters. The ribose transporter system of strain RA is made up of an ATP-binding protein (rbsA, AWN76_002990), substrate-binding protein (rbsB, AWN76_002965), and ABC transport permease protein (rbsC, AWN76_002985), these genes were all upregulated. Two permease proteins (AWN76_014015 and 014025) associated with multiple sugar transporter, were also upregulated. The sugar ABC transport system, which involves three proteins (AWN76_013070, 013075, and 013080), was also upregulated when strain RA was cultured on beechwood xylan.

The transported carbon sources will enter central carbon metabolism. Strain RA uses glycolysis, tricarboxylic acid (TCA) cycle, and the pentose phosphate pathway (PPP) (Table 7). When the media was supplemented with beechwood xylan, many of the related genes, especially those in the glycolysis pathway and TCA cycle, were downregulated (Table 7). The downregulation of these genes indicated that strain RA was tuning down its carbon metabolism during the bacterium harvesting. Data showed that many GHs were upregulated when cells were cultivated in MB+xylan. Furthermore, several types of sugars and ribose transporters were also upregulated. Therefore, it is unclear why the tuning down of carbon metabolism was observed in the transcriptomics analysis when sugars are likely to be abundant inside the cell.

### 3.9. Possible Role of R. sacchariphilus Strain RA in the Environment

As stated earlier, *R. sacchariphilus* strain RA was isolated from a saline hot spring located on an island. At the time of sampling, the temperature of the site was around 40−50 °C and with a pH of 7.1. An influx of ocean and groundwater feeds into the hot spring. The Ayer Hangat hot spring had a high C:N ratio of 5.0, which indicated that the site contains carbon sources [37]. Carbon sources can be contributed by both particulate and dissolved organic carbons [74]. Microorganisms do not readily use particulate carbon polymers for growth. Therefore, bacteria need to produce different enzymes to degrade them. Strain RA harbors almost all enzymes for degrading lignocellulose; however, it is deficient in exoglucanase and effective lignin-acting enzymes. Both groups of enzymes are essential in destroying the protectant layer of plant litter. Strain RA alone is not able to degrade plant litter completely. Therefore, it is anticipated that the bacterium interacts with other microorganisms for such action. Many reported species from the Ayer Hangat hot spring microbiome are good lignocellulose degraders. These include members from the families *Geobacteraceae*, *Dictyoglomaceae*, *Ignavibacteriaceae*, and *Thermoanaerobacterales* [37]. Strain RA and these microorganisms might co-decompose plant litter in free-living form, or presumably more effectively as multi-species biofilms attached to the particulate plant fragments.

According to our earlier metagenomic analysis, strain RA is a minority constituent of the microbial community as it constituted <0.001% of the whole population [37]. The recorded growth rate of strain RA is considered slow in the enriched medium marine broth. Therefore, we hypothesized that the bacterium propagates even slower in situ. Examples of dominant taxa in the hot spring were *Jhaorihella*, *Lyngbya*, *Maritimibacter*, *Roseibacterium*, *Cyanobacterium*, *Vibrio*, *Muricauda*, *Hahellaceae*, *Geothermobacter*, and others; and the majority of these taxa grow faster ex situ [37]. Based on the list of CAZymes genes and the activities of the marker enzymes, strain RA is not a good degrader of plant biomass compared to that of other microorganisms in the hot spring. Furthermore, the performance of strain RA may be relatively low compared to the close relatives *Rhodothermus marinus* (DSM 4252) and *Rhodothermus marinus* SG0.5JP17-172 [24,25,26,27,28,29]. Nevertheless, strain RA had enhanced enzymatic activities, showed better growth, and higher transcriptomic responses toward xylan as the carbon source. Accumulated findings from this project imply that strain RA plays a minor role in the decomposition of hemicellulose fraction of an environmental plant litter.

## 4. Conclusions

Strain RA harbors more than 50 GH genes. We had previously examined the enzymology aspect of xylanases XynRA1 and XynRA2 [31,32]. While the majority of other hydrolases exhibit low similarity to previously described GHs, they might warrant further study. For instance, these enzymes can be used as cocktail formulation for the bioprocessing of lignocellulosic biomass at elevated temperatures. For the first time, the transcriptomic response of *R. sacchariphilus* strain RA cultivated on beechwood xylan was reported. A total of 1122 genes were upregulated, and 1039 genes were downregulated. Many upregulated sequences are associated with hydrolase enzymes, transportation, and membrane components synthesis. Introducing xylan into the culture media induces the production of hemicellulases. In addition, xylan also enhanced the yield of cellulolytic enzymes, which was probably due to the co-expression by the same promoter located adjacent to genes encoding cellulases and hemicellulases. Therefore, xylose and xylan are likely universal inducers for a broad range of hydrolytic enzymes in strain RA. A single time point rather than a time-course transcriptome study was performed in this research. Future analysis of a time-course transcriptomic response may provide additional insights. The detailed in situ response of strain RA in nature remains unclear and may be far more complicated than is currently recognized. The reaction of strain RA in an environmental consortium in the decomposition of plant biomass may be a new research subject.

## Figures and Tables

**Table 1:** The basic information of *Rhodothermales* members.

Strain ^1^	Origin	Opt. Temp; Opt. pH; Opt. NaCl	Hydrolytic Activity ^2^			Genome Size (Mb)/Status	16S rRNA ^3^ (%)	ANI ^4^ (%)	Taxonomy Ref. ^5^	Cellulase/Hemicellulase Ref. ^6^
			A	C	H					
RA	Saline hot spring	50 °C; pH 7; 2% (*w*/*v*)	+	+	+	4.65 (Complete)	100	100	[30]	[31,32]
Rssac	Sea port	55 °C; pH 7; 2–4% (*w*/*v*)	ND	+	ND	4.81 (Complete)	99.3	96.2	[13]	NA
Rmar	Hydrothermal vent	65 °C; pH 6.5–7; 2% (*w*/*v*)	+	+	+	3.39 (Complete)	87.5	73.3	[7]	[24,28,29,33,34,35]
Srub	Saltern crystallizer pond	37–47 °C; pH 6.5–8; 20–30% (*w*/*v*)	+	ND	ND	3.59 (Complete)	85.6	71.9	[14]	NA
Sira	Salt Lake	37 °C; pH 7.5; 17% (*w*/*v*)	-	ND	ND	3.41 (Draft)	83.1	70.0	[15]	NA
Slon	Dead/Red sea water	37–46 °C; pH 6.5–8.5; 10–12% (*w*/*v*)	+	ND	ND	3.19 (Draft)	85.7	71.5	[18]	NA
Lhalo	Marine solar saltern	37–42 °C; pH 7.5–8; 6–8% (*w*/*v*)	+	-	ND	3.73 (Draft)	81.9	69.1	[19]	NA
Lsali	Marine solar saltern	40 °C; pH 7.5–8; 8–12% (*w*/*v*)	+	-	ND	4.41 (Draft)	82.4	69.3	[20]	NA
Npro	Hypersaline alkaline lake	37 °C; pH 9.5–9.8; 14.6–17.5% (*w*/*v*)	+	-	-	ND	83.8	ND	[21]	NA
Rcmar	Sea water	20–30 °C; pH 5–9; 2% (*w*/*v*)	ND	ND	ND	4.43 (Draft)	81.5	69.5	[16]	NA
Rvmar	Deep sea water	25–30 °C; pH 6–8; 1–5% (*w*/*v*)	ND	ND	ND	4.98 (Draft)	81.3	69.9	[17]	NA

^1^ Abbreviation for bacteria strains – RA: strain RA, Rssac: *Roseithermus sacchariphilus*, Rmar: *Rhodothermus marinus* DSM 4252, Srub: *Salinibacter ruber* (DSM 13855), Sira: *Salinivenus iranica* CB7, Slon: *Salisaeta longa* (DSM 21114), Lhalo: *Longimonas halophila*, Lsali: *Longibacter salinarum*, Npro: *Natronotalea proteinilytica*, Rcmar: *Rubricoccus marinus*, and Rvmar: *Rubrivirga marina*. ^2^ Hydrolytic Activity – A: Amylolytic activity tested against starch, C: Cellulolytic activity tested against carboxymethyl cellulose, H: Hemicellulolytic activity tested against xylan; symbols ‘-’: negative result; ‘+’: positive result, ND: Not Determined. ^3,4^ 16S rRNA similarity and Average Nucleotide Identity (ANI) as compared to *R. sacchariphilus* strain RA. Genome-to-genome ANI values were determined using OrthoANI [36]. *Natronotalea proteinilytica*’s genome is unavailable. ^5^ Taxonomy related reports for the first isolated strain. ^6^ Examples of articles on the discovery of cellulase or hemicellulase. NA: Not Available.

**Table 2:** Statistics information of the six RNA-seq datasets.

Sample ^1^		Raw Reads	Q20 (%) ^2^	Clean Reads ^3^	Total Mapped Reads ^4^	Uniquely Mapped (%)	Multiple Mapped (%)
MB	1	21,980,340	97.81	21,497,336	21,402,185 (99.56%)	98.40	1.16
	2	22,412,010	97.91	21,857,496	21,728,678 (99.41%)	98.18	1.23
	3	21,996,694	97.86	21,478,716	21,381,885 (99.55%)	98.36	1.19
MB+xylan	1	23,769,554	97.61	23,307,532	23,022,398 (98.78%)	97.69	1.09
	2	25,168,712	97.58	24,610,454	24,377,051 (99.05%)	97.96	1.09
	3	22,425,294	97.69	21,935,340	21,597,320 (98.46%)	97.30	1.16

^1^ 1, 2, and 3 represent the three biological replicates of each experiment setups for strain RA grown in MB and MB+xylan, respectively. ^2^ Q20 is the setting used for filtering raw reads into clean reads, with Q_Phred_ value set at 20. ^3^ The number of clean reads obtained from Q20 filtering. ^4^ The total clean reads or percentage that can align with the complete genome.

**Table 3:** Fold change of transcriptional factors identified in strain RA.

Gene ID and Annotated Names	FC and Gene Regulation Status
AraC family (regulates carbon catabolism, stress responses, and virulence)	
AWN76_004845 AraC family transcriptional regulator	~
AWN76_005395 AraC family transcriptional regulator	1.52➚
AWN76_009110 AraC family transcriptional regulator	0.26➘
AWN76_012545 AraC family transcriptional regulator	~
AWN76_014955 AraC family transcriptional regulator	~
DeoR family (regulates sugar catabolism, regulates aga operon for n-acetyl galactosamine transport and metabolism)	
AWN76_014420 transcriptional regulator AgaR	1.94➚
AWN76_014295 DeoR family transcriptional regulator	1.93➚
LacI family (regulates sugar/lactose catabolism)	
AWN76_006400 LacI family transcriptional regulator	1.54➚
AWN76_010965 LacI family transcriptional regulator	~
AWN76_004120 LacI family transcriptional regulator	2.02➚
AWN76_016215 LacI family transcriptional regulator	~
AWN76_013990 LacI family transcriptional regulator	2.49➚
AWN76_003445 LacI family transcriptional regulator	0.56➘
AWN76_012325 LacI family transcriptional regulator	~
GntR family (regulates carbohydrate transport and metabolism; transcriptional repressor for pyruvate dehydrogenase complex)	
AWN76_002320 GntR family transcriptional regulator	0.37➘
AWN76_018420 transcriptional regulator PdhR	0.39➘
AWN76_008220 transcriptional regulator PdhR	0.41➘
AWN76_003490 GntR family transcriptional factor	2.37➚

Footnote: FC: Fold change; ➚: Upregulated and statistically significant; ➘: downregulated and statistically significant; ~: Non-Differentially Expressed Genes (DEGs), FC is negligible, or value is statistically insignificant.

**Table 4:** Fold change of genes related to chemotaxis and flagella assembly.

Gene ID and Annotated Names	FC and Gene Regulation Status
**Chemotaxis pathway**	
AWN76_009955 methyl-accepting chemotaxis protein MCP	1.90➚
AWN76_011645 methyl-accepting chemotaxis protein MCP	~
AWN76_016375 methyl-accepting chemotaxis protein MCP	2.98➚
AWN76_017480 methyl-accepting chemotaxis protein MCP	~
AWN76_017530 methyl-accepting chemotaxis protein MCP	3.29➚
AWN76_017495 sensor kinase CheA	2.32 ➚
AWN76_017520 chemotaxis protein methyltransferase CheR	0.45➘
AWN76_017540 purine-binding chemotaxis protein CheW	~
AWN76_008370 chemotaxis protein CheY	2.29 ➚
AWN76_016800 chemotaxis protein CheY	3.70➚
AWN76_017505 chemotaxis protein CheY	~
AWN76_017515 chemotaxis protein CheY	0.40➘
AWN76_017500 chemotaxis protein CheZ	2.20➚
**Flagella assembly**	
AWN76_013275 flagellar motor protein MotB	~
AWN76_017405 RNA polymerase sigma factor FliA	0.51➘
AWN76_017420 flagellar biosynthesis protein FlhF	~
AWN76_017415 flagellar protein FliS	2.61➚
AWN76_017425 flagellar biosynthesis protein FlhA	~
AWN76_017430 flagellar biosynthetic protein FlhB	~
AWN76_017435 flagellar biosynthetic protein FliR	2.31➚
AWN76_017440 flagellar biosynthetic protein FliQ	~
AWN76_017445 flagellar biosynthetic protein FliP	0.65➘
AWN76_017455 flagellar motor switch protein FliN	3.20➚
AWN76_017460 flagellar hook-basal body complex protein FliM	~
AWN76_017465 flagellar FliL protein	~
AWN76_017470 flagellar motor protein MotB	~
AWN76_017475 flagellar motor protein MotA	~
AWN76_017545 flagellar hook protein FlgE	~
AWN76_017555 flagellar hook assembly protein FlgD	~
AWN76_017575 flagellum-specific ATP synthase	3.70➚
AWN76_017580 flagellar assembly protein FliH	3.59➚
AWN76_017585 flagellar motor switch protein FliG	3.53➚
AWN76_017595 flagellar hook-basal body complex protein FliE	~
AWN76_017605 flagellar basal body rod protein FlgC	~
AWN76_017610 flagellar biosynthesis protein FlgB	~
AWN76_017635 flagellar hook protein FliD	0.59➘
AWN76_017640 flagellar protein FliS	~
AWN76_017650 flagellin FliC	5.20➚
AWN76_017655 flagellin FliC	2.15➚
AWN76_017660 flagellin FliC	~
AWN76_017675flagellar hook-associated protein 3 FlgL	1.91➚
AWN76_017680 flagellar hook-associated protein FlgK	2.00➚
AWN76_017695 flagellar basal body P-ring protein FlgI	2.82➚
AWN76_017700 flagellar basal body L-ring protein FlgH	~
AWN76_017705 flagella basal body P-ring formation protein FlgA	~
AWN76_017710 flagellar basal-body rod protein FlgG	~
AWN76_017715 flagellar basal-body rod protein FlgF	0.62➘

Footnote: FC: Fold change; ➚: Upregulated and statistically significant; ➘: downregulated and statistically significant; ~: Non-DEGs, FC is negligible, or value is statistical insignificant.

**Table 5:** Fold change of (hemi-)cellulolytic genes encoded glycosyl hydrolases (GHs).

	Gene ID and Annotated Names	FC and Gene Regulation Status
**Family**	**Cellulolytic GHs**	
GH3	AWN76_006445 β-glucosidase ^(1)^	5.30➚
GH44	AWN76_008195 hypothetical protein ^(2)^	1.45➚
GH3	AWN76_008215 β-glucosidase ^(3)^	3.43➚
GH9	AWN76_008290 cellulase ^(4)^	1.99➚
GH5	AWN76_009395 glycoside hydrolase ^(5)^	1.78➚
GH9	AWN76_010685 glycoside hydrolase family 9 ^(6)^	~
**Family**	**Hemicellulolytic GHs**	
GH78	AWN76_002810 α-l-rhamnosidase	2.53➚
GH92	AWN76_002955 α-1,2-mannosidase	~
GH10	AWN76_003690 endo-1,4-β-xylanase ^(7)^	~
GH31	AWN76_004235 glycoside hydrolase family 31	~
GH31	AWN76_008190 α-xylosidase	~
GH10	AWN76_008205 endo-1,4-β-xylanase ^(8)^	1.66➚
GH67	AWN76_008230 α-glucuronidase ^(9)^	1.70➚
GH106	AWN76_008320 α-l-rhamnosidase ^(10)^	3.28➚
GH78	AWN76_009025 α-l-rhamnosidase	1.60➚
GH16	AWN76_009940 glycoside hydrolase family 16	~
GH29	AWN76_010630 α-l-fucosidase	~
GH78	AWN76_012010 α-l-rhamnosidase	2.61➚
GH43	AWN76_012335 β-xylosidase ^(11)^	~
GH51	AWN76_012350 α-d-arabinofuranosidase ^(12)^	2.74➚
GH113	AWN76_013895 β-mannase ^(13)^	3.31➚
GH130	AWN76_014035 glycosidase	4.00➚
GH130	AWN76_014055 glycosidase	3.90➚
GH2	AWN76_014570 glycoside hydrolase family 2	~
GH154	AWN76_017060 hypothetical protein	~
GH53	AWN76_017855 endo-1,4-β-galactanase ^(14)^	2.43➚

Footnote: FC: Fold change; ➚: Upregulated and statistically significant; ~: Non DEGs, FC is negligible, or value is statistical insignificant. ^(1)–(14)^ The annotation ending with supercripted bracket numbering refers to the represented enzymes showed in Figure 6.

**Table 6:** Fold change of putative auxiliary activities enzymes (AAs) in strain RA.

	Gene ID and Annotated Names	FC and Gene Regulation Status
**Family**	**AAs**	
AA3	AWN76_001955 GMC family oxidoreductase	0.44➘
AA3	AWN76_003120 GMC family oxidoreductase	0.43➘
AA12	AWN76_005825 sorbosone dehydrogenase	~
AA3	AWN76_007025 GMC family oxidoreductase	1.88➚
AA3	AWN76_007050 patatin-like phospholipase family protein	2.70➚
AA12	AWN76_011490 sorbosone dehydrogenase	~
AA3	AWN76_011750 GMC family oxidoreductase	0.60➘
AA2	AWN76_014060 catalase/peroxidase HPI	0.36➘

Footnote: FC: Fold change; ➚: Upregulated and statistically significant; ➘: downregulated and statistically significant; ~: Non-DEGs, FC is negligible or value is statistical insignificant.

**Table 7:** Fold change of genes related to carbon metabolism when strain RA was cultivated in beechwood xylan.

Gene ID and Annotated Names	FC and Gene Regulation Status
**Glycolysis pathway**	
AWN76_000290 triose-phosphate isomerase	1.49➚
AWN76_000410 pyruvate kinase	0.49➘
AWN76_001275 fructose-bisphosphate aldolase	0.15➘
AWN76_003230 phosphoglycerate kinase	~
AWN76_003235 glyceraldehyde-3-phosphate dehydrogenase	0.26➘
AWN76_004260 enolase	0.49➘
AWN76_004270 glucokinase	~
AWN76_005185 glucose/mannose-6-phosphate isomerase	~
AWN76_005345 6-phosphofructokinase	~
AWN76_007495 phosphoglycerate mutase	~
AWN76_007770 fructose-1,6-bisphosphatase I	0.56➘
AWN76_009045 6-phosphofructokinase	~
AWN76_012860 glucose/mannose-6-phosphate isomerase	~
AWN76_013745 6-phosphofructokinase	~
AWN76_014560 galactose mutarotase	0.65➘
AWN76_017730 polyphosphate glucokinase	~
AWN76_018330 phosphoglucomutase	~
**Tricarboxylic acid (TCA) cycle**	
AWN76_001060 pyruvate-ferredoxin/flavodoxin oxidoreductase	3.04➚
AWN76_003860 class II fumarate hydratase	0.39➘
AWN76_004160 malate dehydrogenase	0.22➘
AWN76_004295 succinyl-CoA synthetase β subunit	0.47➘
AWN76_004595 dihydrolipoyl dehydrogenase	0.39➘
AWN76_004770 NADP-dependent isocitrate dehydrogenas	~
AWN76_006135 isocitrate dehydrogenase (NAD+)	0.59➘
AWN76_006890 pyruvate dehydrogenase E2 component	0.56➘
AWN76_006895 pyruvate dehydrogenase E1 subunit β	0.27➘
AWN76_006900 pyruvate dehydrogenase E1 subunit α	0.16➘
AWN76_006920 NADP-dependent isocitrate dehydrogenase	0.19➘
AWN76_008605 2-oxoglutarate dehydrogenase E2 component	0.46➘
AWN76_009515 succinate-CoA ligase subunit α	0.43➘
AWN76_009950 2-oxoglutarate dehydrogenase E1 component	0.40➘
AWN76_011245 citrate synthase	0.57➘
AWN76_011250 succinate dehydrogenase cytochrome b subunit	0.37➘
AWN76_011255 succinate dehydrogenase	0.52➘
AWN76_011260 succinate dehydrogenase flavoprotein subunit	0.51➘
AWN76_011265 succinate dehydrogenase iron–sulfur subunit	0.46➘
AWN76_014835 citrate synthase	0.28➘
AWN76_014900 2-oxoglutarate/2-oxoacid ferredoxin oxidoreductase subunit β	0.64➘
AWN76_017390 pyruvate dehydrogenase E2 component	~
AWN76_018190 aconitate hydratase	0.35➘
**Pentose phosphate pathway (PPP)**	
AWN76_002240 6-phosphogluconolactonase	2.36➚
AWN76_002245 glucose-6-phosphate dehydrogenase	~
AWN76_002250 6-phosphogluconate dehydrogenase	2.72➚
AWN76_005185 glucose/mannose-6-phosphate isomerase	~
AWN76_005350 6-phosphogluconate dehydrogenase	0.47➘
AWN76_006030 transketolase	~
AWN76_008645 ribose-phosphate pyrophosphokinase	0.37➘
AWN76_008995 fructose-6-phosphate aldolase	0.40➘
AWN76_010760 d-arabino 3-hexulose 6-phosphate aldehyde lyase	0.62➘
AWN76_012150 ribulose-phosphate 3-epimerase	~
AWN76_012860 glucose-6-phosphate isomerase	~
AWN76_016655 ribose-5-phosphate isomerase A	1.86➚
**Other genes involved in carbon metabolism**	
AWN76_000185 aminomethyltransferase	0.64➘
AWN76_002260 bifunctional methylenetetrahydrofolate dehydrogenase/methenyltetrahydrofolate cyclohydrolase	~
AWN76_002310 phosphoenolpyruvate carboxykinase (ATP)	0.34➘
AWN76_002820 methylmalonyl-CoA mutase	~
AWN76_003760 glycerate 2-kinase	2.74➚
AWN76_003920 d-3-phosphoglycerate dehydrogenase/2-oxoglutarate reductase	2.04➚
AWN76_004480 phosphoenolpyruvate carboxylase	0.62➘
AWN76_004595 dihydrolipoyl dehydrogenase	0.39➘
AWN76_005090 acetyl-CoA carboxylase carboxyltransferase subunit β	0.26➘
AWN76_005450 zinc-binding alcohol dehydrogenase family protein	0.63➘
AWN76_005870 3-hydroxyacyl-CoA dehydrogenase/enoyl-CoA hydratase family protein	0.51➘
AWN76_006190 acetate-CoA ligase	0.37➘
AWN76_006195 acetyl-CoA C-acyltransferase	
AWN76_006780 acetyl-coenzyme A synthetase	1.98➚
AWN76_007665 glycine cleavage system protein GcvH	0.39➘
AWN76_007670 acetyl-CoA carboxylase, biotin carboxylase	~
AWN76_007675 acetyl-CoA carboxylase, biotin carboxyl carrier protein	0.53➘
AWN76_008645 ribose-phosphate pyrophosphokinase	0.37➘
AWN76_009505 phosphoglycerate dehydrogenase	~
AWN76_009860 aldehyde dehydrogenase	~
AWN76_009995 enoyl-CoA hydratase	~
AWN76_010345 methylenetetrahydrofolate reductase	0.48➘
AWN76_010475 acetyl-CoA carboxylase carboxyltransferase subunit α	~
AWN76_010955 bifunctional 4-hydroxy-2-oxoglutarate aldolase/2-dehydro-3-deoxy-phosphogluconate aldolase	2.34➚
AWN76_011330 acyl-CoA carboxylase subunit β	~
AWN76_012245 l-lactate dehydrogenase	~
AWN76_013500 Threonine dehydratase	2.04➚
AWN76_014285 glycine dehydrogenase (aminomethyl-transferring)	0.50➘
AWN76_014810 serine hydroxymethyltransferase	0.57➘
AWN76_016000 aldehyde dehydrogenase family protein	2.22➚
AWN76_016690 Glutamate dehydrogenase	0.33➘
AWN76_017295 methylmalonyl-CoA epimerase	~
AWN76_018450 3-phosphoserine/phosphohydroxythreonine transaminase	0.42➘

Footnote: FC: Fold change; ➚: Upregulated and statistically significant; ➘: downregulated and statistically significant; ~: Non-DEGs, FC is negligible, or value is statistical insignificant.
